# Healthcare associated infection and its risk factors among patients admitted to a tertiary hospital in Ethiopia: longitudinal study

**DOI:** 10.1186/s13756-017-0298-5

**Published:** 2018-01-05

**Authors:** Solomon Ali, Melkamu Birhane, Sisay Bekele, Gebre Kibru, Lule Teshager, Yonas Yilma, Yesuf Ahmed, Netsanet Fentahun, Henok Assefa, Mulatu Gashaw, Esayas Kebede Gudina

**Affiliations:** 1 School of Medical laboratory Science, Institute of Health, School of Medical laboratory Science, Jimma University, P.O. Box 1368, Jimma, Ethiopia; 2 Department of paediatrics and child health, Jimma University, Jimma, Ethiopia; 3 Department of ophthalmology, Jimma University, Jimma, Ethiopia; 4 Department of Surgery, Jimma University, Jimma, Ethiopia; 5 Department of Obstetrics and Gynaecology, Jimma University, Jimma, Ethiopia; 6 Department of Health education and behavioural health, Jimma University, Jimma, Ethiopia; 7 Department of Epidemiology, Jimma University, Jimma, Ethiopia; 8 Department of Internal Medicine, Jimma University, Jimma, Ethiopia; 9 WHO-TDR clinical research former fellow at AERAS Africa and Rockville, Rockville, MD USA

**Keywords:** Health-care associated infection, Nosocomial infection, Jimma, Ethiopia, Africa

## Abstract

**Background:**

Healthcare associated infection (HAI) is alarmingly increasing in low income settings. In Ethiopia, the burden of HAI is still not well described.

**Methods:**

Longitudinal study was conducted from May to September, 2016. All wards of Jimma University Medical Centre were included. The incidence, prevalence and risk factors of healthcare associated infection were determined. A total of 1015 admitted patients were followed throughout their hospital stay. Biological specimens were collected from all patients suspected to have hospital aquired infection. The specimens were processed by standard microbiological methods to isolate and identify bacteria etiology. Clinical and laboratory data were collected using structured case report formats.

**Results:**

The incidence rate of hospital acquired infection was 28.15 [95% C.I:24.40,32.30] per 1000 patient days while the overall prevalence was 19.41% (95% C.I: (16.97–21.85). The highest incidence of HAI was seen in intensive care unit [207.55 (95% C.I:133.40,309.1) per 1000 patient days] and the lowest incidence was reported from ophthalmology ward [0.98 (95% C.I: 0.05,4.90) per 1000patient days]. Among patients who underwent surgical procedure, the risk of HAI was found to be high in those with history of previous hospitalization (ARR = 1.65, 95% C.I:1.07, 2.54). On the other hand, young adults (18 to 30-year-old) had lower risk of developing HAI (ARR = 0.54 95% C.I: 0.32,0.93) Likewise, among non-surgical care groups, the risk of HAI was found to be high in patients with chest tube (ARR = 4.14, 95% C.I: 2.30,7.46), on mechanical ventilation (ARR = 1.99, 95% C.I: 1.06,3.74) and with underlying disease (ARR = 2.01, 95% C.I: 1.33,3.04). Furthermore, hospital aquired infection at the hosoital was associated with prolonged hospital stay [6.3 more days, 95% C.I: (5.16,7.48), *t* = 0.000] and increased in hospital mortality (AOR, 2.23, 95% CI:1.15,4.29).

**Conclusion:**

This study revealed high burden and poor discharge outcomes of healthcare associated infection at Jimma University Medical Centre. There is a difference in risk factors between patients with and without surgery. Hence, any effort to control the observed high burden of HAI at the hospital should consider these differences for better positive out put.

**Electronic supplementary material:**

The online version of this article (10.1186/s13756-017-0298-5) contains supplementary material, which is available to authorized users.

## Background

Healthcare associated infection (HAI) is localized or systemic condition resulting from adverse reaction to the presence of infectious agent or its toxins acquired from health care settings that was not incubating or symptomatic at the time of admission to the healthcare facility [1]. It accounts for a large proportion of damages caused by healthcare processes in both developed countries and low income settings [2–4]. HAI is increasingly becoming a major global public health problem posing great threat to patient safety and wellbeing of healthcare providers [5, 6].

HAIs contribute to increased morbidity and mortality [7, 8], excess health care cost [9, 10] and prolonged hospital stay [11]. It has a far reaching consequence to the public resulting in widespread occurrence of multidrug resistant pathogens in hospital settings [12, 13] and dissemination of emerging and re-emerging infections to healthcare providers and the community [14, 15].

HAI affects about 7.6% of patients in regular wards [16] and at least half of those admitted to intensive care units (ICU) [17] in developed countries. The magnitude of the problem in the low-income settings remains largely unknown and in most cases underestimated due to the complex nature of its diagnosis and lack of proper surveillance [18, 19]. Studies conducted in low-income settings showed that hospital-wide prevalence of HAI is about 15.5 per 100 patients [18] which is much higher than reports from Europe [20] and North America [21]. As a matter of fact, most of the global reports of HAIs are focused on prevalence and there are only very few reports about the incidence rate of HAIs.

In Ethiopia, few HAIs studies focused on point prevalence are available since 1988 [22, 23]. Many of these were limited to postoperative HAIs [22–25] with estimated prevalence of 11% to 36% [24–27]. Surgical site, urinary tract and blood stream infections were found to be commonest forms [24–26]. The type of surgery, patients’ underlying medical conditions and the type of the ward were found to be important factors associated with increased risk of HAI in Ethiopia [22, 23, 28, 29].

At least 50% of HAIs are preventable with current evidence based interventions [19, 30]. Despite this fact, they remain great threat to quality of healthcare delivery system worldwide and even more so in low-income settings. Overcrowding and limited number of health task force in hospitals result in inadequate infection control practices. On top of these, lack of infection control policies, guidelines and trained professionals make the problem even worse [18, 19].

To the best of our knowledge, there is no single report about the incidence rate of HAIs from Ethiopia before. All previously published studies were estimted the point prevalence of HAIs based on a data collected from one or two wards of respective hospitals [22–27]. In the current study, we tried to estimate the incidence rate of HAI at tertiary hospital in southwest Ethiopia. The study also aimed to provide a comprhensive hospital level prevalence data to overcome the limitations in the previous reports from the country as most of them were limited to few wards and units.

## Methods

### Setting

The study was conducted at Jimma University Medical Centre (JUMC) in southwest Ethiopia. It is the only referral and teaching medical centre in the region serving over 15 million people. The annual average admission of the centre is over 15,000 patients (https://www.ju.edu.et/jimma-university-specialized-hospital-jush).

### Study design and study population

A hospital based longitudinal study design was conducted from May 1 to September 30, 2016. All patients admitted to ICU, gynaecology & obstetrics, paediatrics, surgical, ophthalmology, and medical wards with no evidence of bacterial infections at admission were included in the study.

### Data collection procedures

Written consent was obtained from each participant prior to commencing any study procedures. For pediatric participants consent from the parents/ legal gardians together with an assent from the child was obtained. For patients in comma, their closest relatives were consented. Socio-demographic and clinical data were collected by structured questioner (Additional file 1). They were followed first for 48 h. Patients who have developed any form of infection within 48 h of admission and/or had asymptomatic bacteriuria were excluded. All the rest of the patients were followed until discharge for the occurrence of HAI, progress or death by attending physician and research staffs. Occurance of HAI was confirmed by sinior specilists of this research team members working in respective wards. All clinical data from all participants were registered on case report form (CRF).

Biological specimens were collected from those participants suspected to have HAI. The specimens were investigated by microbiological methods following WHO manual to isolate the culprit bacteria [31]. Furthermore, blood culture was done by using BD BACTEC™ FX40 blood culture system as per manufacturers’ instruction.

### Definition of outcome variables

The primary outcome variable was occurrence of infection after 48 h of hospitalization in a patient otherwise not having symptomatic or incubating infection on hospital admission. The following are definitions used in this study for specific type of HAI (*Adopted from* Centre for disease control/National Health care Safety Network (*CDC/NHSN) surveillance definition for health care–associated infection* [1] with slight modification):

#### Blood stream infection

Patient with any of the following signs and symptoms: fever (>38 °C), Chills/rigours or hypotension and at least one positive blood culture not related to contamination.

### Healthcare associated pneumonia

Respiratory symptoms with at least two of the following signs and symptoms appearing during hospitalization: cough, purulent sputum, new infiltrate on chest radiograph consistent with infection.

### Surgical site infection

Any purulent discharge, abscess, or spreading cellulitis at the surgical site during the month after the operation.

### Urinary tract infection

Mid-stream urine cultures with ≥10^5^ colony forming units (CFU) and catheter urine with ≥10^2^ CFU/ml with no more than 2 species of microorganisms isolated OR positive dipstick for leukocyte esterase OR pyuria (≥10 white blood cells /high power field) of clean catch urine in a patients with or without signs and symptoms in the presence or absence of recent urinary catheterization.

### Data management and analysis

The collected data were checked for completeness and then entered to EpiData version 3.1. Then, it was exported to STATA® version 10.0. (College Station, TX: StataCorp LP) and SPSS® Statistics for Windows, Version 20.0 (Armonk, NY: IBM Corp) for analysis. Incidence and prevalence of nosocomial infection was determined at the confidence interval of 95%. Chi-square test was done to identify factors associated with HAIs first. Then bi-variate and multivariate logistic regression was used to depict the risk ratio (RR) and adjusted risk ratio (ARR) to identify factors with increased risk of HAIs. A *p*-value of <0.05 was used as level of statistical significance.

#### Data quality control

All data collectors were trained on basic procedures of data collection. Data collection was closely supervised by principal investigators. To ensure quality of clinical data and specimen, all clinical information were collected by trained treating physicians and research nurses in each ward. Clinical specimens were collected and processed as per Standard Operating procedures (SOPs) for isolation and identification of bacteria. Qualities of prepared culture plates and broth were checked for their sterility and performance as per the SOP. All laboratory tests were performed by qualified laboratory professionals after one day orientation training.

## Results

### Socio-demographic characteristics of the participants

A total of 1069 hospitalized patients were enrolled in this study. However, 54 participants showed signs of infection and/or asymptomatic bacteriuria within the first 48 h and were excluded from the study. The rest 1015 were followed for the occurrence of hospital acquired infection until discharge or death. From these, 23 participants were excluded from final analysis due to incomplete data set. A total of 992 participants were included in the final analysis.

About 55% of the participants were female. The mean age in years was 34.12 (SD ± 16.86). Almost half (49.20%) of the participants were younger than 30 years of age (Table 1). Obstetrics and gynaecology, surgical, and medical wards constituted for around a quarter of participants each. Furthermore, 22 (2.22%) of the participants were included from intensive care unit (ICU) (Table 1). More than half, 510 (51.41%) of the participants underwent surgical procedures during the current admission. From these, 244 (47.94%) had emergency surgical procedures of which majority, 149 (61.07%), had Caesarean section.

**Table 1:** Background characteristics of the patients admitted to Jimma University Medical Centre, Ethiopia

Characteristic	Frequency	Proportion in % (95% CI)
Sex		
Male	443	44.66 (41.41.56–47.76)
Female	549	55.34 (52.24–58.44)
Age in Years		
< 18	147	14.82 (12.60–17.03)
18–30	341	34.38 (31.41–37.33)
31–45	269	27.12 (24.35–29.88)
> 45	235	23.69 (21.04–26.34)
Residency		
Urban	361	36.39 (33.39–39.39
Rural	631	63.861 (60.61–66.61)
Occupation^a^		
Farmer	371	37.40 (34.38–40.41)
Employed	148	14.92 (12.70–17.14)
Student	108	10.89 (8.95–12.82)
Merchant	102	10.28 (8.39–12.18)
Daily labourer	84	8.47 (6.73–10.20)
Others	179	18.08 (15.65–20.44)
Marital Status^b^		
Married	700	70.56 (67.72–73.41)
Single	180	24.09 (21.43–26.76)
Divorced	27	2.72 (1.71–3.74)
Widowed	26	2.62 (1.63–3.62)
Education		
Illiterate	448	45.16 (42.06–48.26)
Read & write	127	12.80(10.72–14.89)
Elementary school	151	15.22 (12.98–17.46)
High school	93	9.38 (7.56–11.19)
Diploma	81	8.17 (6.46–9.87)
First degree	70	7.06 (5.46–8.65)
Above first degree	22	2.22 (1.30–3.14)
Ward of admission		
Obstetrics and Gynaecology	282	28.43 (25.62–31.24)
Surgical	253	25.50 (22.79–28.22
Medical	223	22.48 (19.88–25.08)
Ophthalmology	153	15.42 (13.17–17.67)
Paediatrics	59	5.95 (4.47–7.42)
ICU	22	2.22 (1.23–3.14)

^a^Parental occupation and education in case of paediatrics, *ICU* intensive care unit

^b^59 paediatrics participants were excluded

Underlying chronic medical conditions were reported by 263 (26.54%) participants. Cardiovascular diseases was the most common co-morbidity identified in 67 (25.48%) participants followed by hypertension, 57 (21.67%) and diabetes mellitus (DM) 39 (14.83%). Severe acute malnutrition, mainly among paediatric patients and HIV infection were also reported in 23 (8.75%) participants each. Twenty (7.60%) of participants were already diagnosed with different forms of cancer and tuberculosis each. Chronic renal failure was also reported from 14 (5.32%) participants, Overall, 76 (7.66%) patients have also reported history of prior hospitalization for either the same as current reason of admission or other ailments.

### Incidence, prevalence and type of health care associated infection

**Fig. 1:**
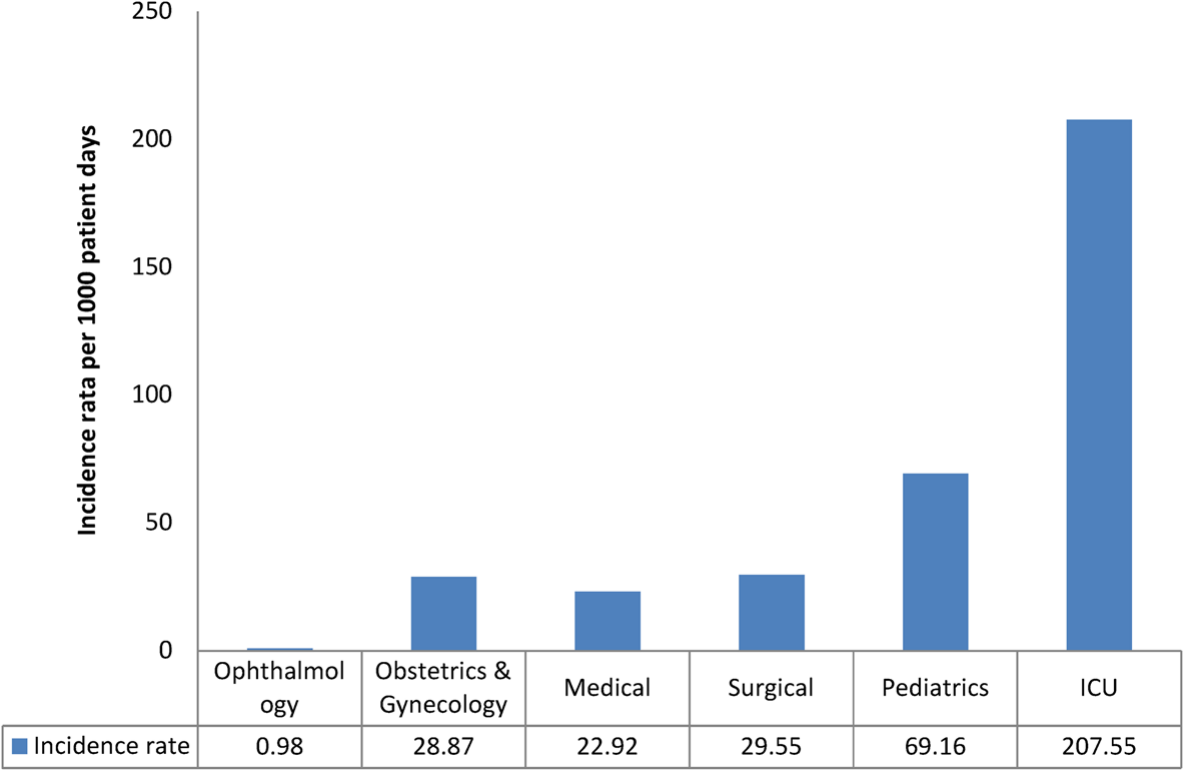
Incidence rate of healthcare associated infection in different wards of admission at Jimma University Medical Centre, Ethiopia

The mean onset of HAI is 4.64 (95% C.I:4.42, 8.86) patient days. The overall incidence rate of HAI at JUMC was 28.15 (95% C.I 24.40, 32.30) per 1000 patient days while the prevalence was 19.41% (195/992) (95% C.I: 16.97, 21.85). Stratification of the incidence by ward of admission revealed significant variability. The highest incidence rate was seen in ICU (207.60, 95% C.I:133.40, 309.10) per 1000 patient days. Conversely, the lowest incidence rate of HAIs was seen in ophthalmology ward (0.98, 95% C.I: 0.05, 4.90) per 1000 patient days. The incidence rate of HAIs in paediatrics and surgical wards were 69.16 (95% C.I:45.30,101.30) and 29.55 (95% C.I: 23.1,37.2) per 1000 patient days respectively (Fig. 1).

**Fig. 2:**
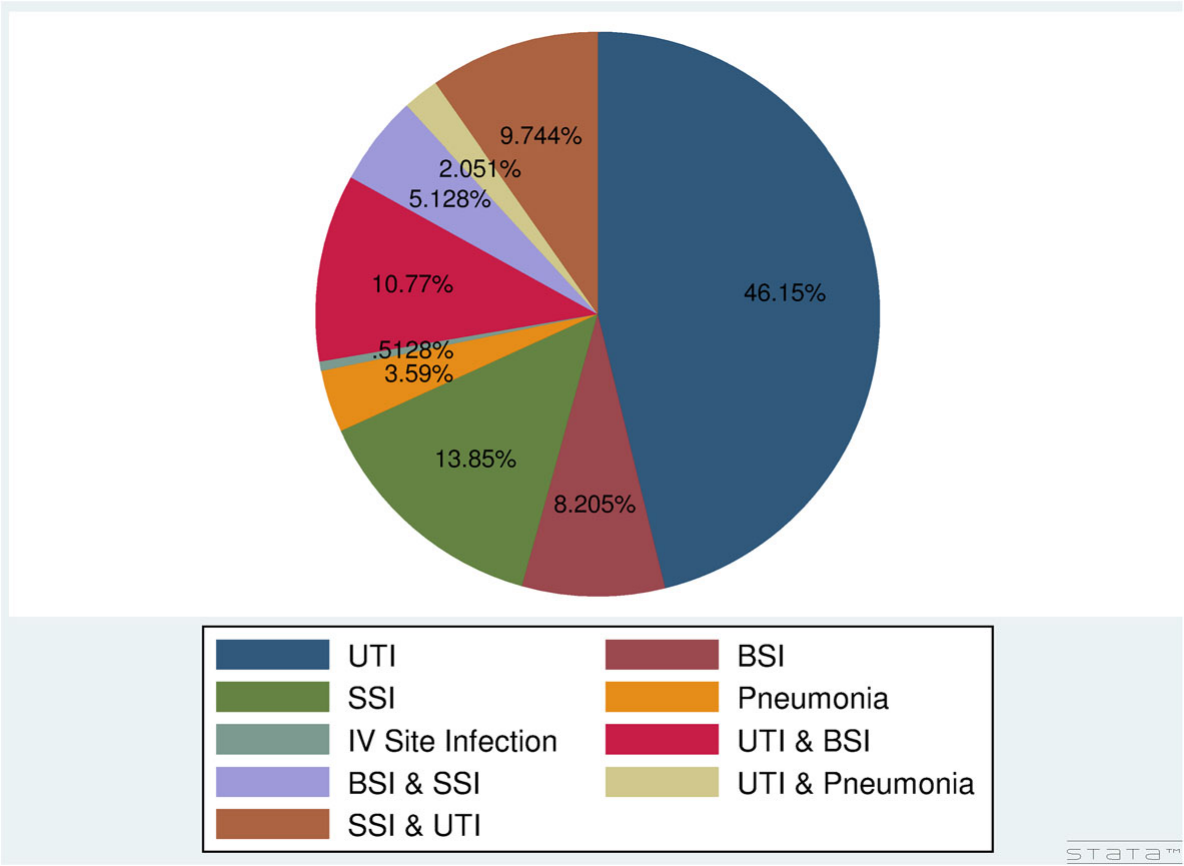
Type of Healthcare associated infection at Jimma University Medical Centre, Ethiopia, where BSI – blood stream infection, IV – intravenous, SSI – surgical site infection, UTI – urinary tract infection

The most frequently observed type of hospital acquired infection was UTI reported in 68.71% of the cases (alone and in combination with other infections) followed by surgical site infection (SSI), 28.72% (alone and in combination with other infections). It is observed that 27.69% of the participants had developed two types of HAI at the same time (Fig. 2).

Clinical samples were collected from 192 (97.46%) participants with HAI to identify causative microorganism. Three requested specimens were missed because participants were not able to give the specimen. Overall, 22 species of bacteria were isolated from 118 specimen which gives 118/192 (61.46%) culture positivity rate. Of these, 16 (72.73%) were Gram negative and (27.27%) were Gram positive bacteria. The most common isolated bacteria were *Escherichia coli* (26.27%), *Klebsella species* (24.58%) and *Staphylococcus aureus* (17.80%).

### Comparison of participants with HAI and without HAI

From the total enrolled 992 participants, 22 of them were admitted to ICU. Furthermore, 50 patients had cataract surgery and were admitted to ophthalmology ward. Most patients admitted to ICU were younger than 24 years of age (59.09%), on mechanical ventilator (57.14%), on NG tube (54.55%), on peripheral IV line (72.73%) and had indwelling urinary catheter (59.09%). However, statistical analysis could not be applied for analysis of risks since all patients admitted to ICU had developed HAIs. On the other hand, only 1 participant with cataract surgery had developed HAI. Again, it is not possible to apply statistical test to analyse risks of HAIs in this ward. Thus, 22 participants admitted to ICU and 50 participants who had cataract surgery were excluded from risk factor analysis.

We have stratified the rest 920 participants in to two groups based on whether they had surgical procedures or not to precisely identify risk factors for those with and without surgery. Accordingly, 450 participants had surgery and grouped as “Surgical care”. The rest 470 participants didn’t have any surgery and grouped as “Non-surgical care”. The demographic and baseline clinical characteristics were compared between patients who developed HAIs and those who did not in each specific group (Table 2).

**Table 2:** Risk of HAI in relation to age, sex, underlying disease and healthcare risk factors among patients with surgical care and without

Variable	Surgical care (*n* = 450)				Non-surgical care (*n* = 470)			
	HAI (%/)***	Total	RR (95% CI)	ARR(95% CI)	HAI (%)	Total	RR(95%CI)	ARR(95% CI)
Age in years								
< 18	20(33.33)	60			20(27.40)	73		
18–30	28(13.53)	207	0.41(0.25,0.67)*	0.54(0.32,0.93)*	19(15,45)	123	0.56(0.32,0.98)	0.61(0.34,1.07
31–45	25(22.94)	109	0.69(0.42,1.13)	0.94(0.56,1.55)	27(18.24)	148	0.67(0.40,1.10)	0.54(0.31,0.93)
> 45	26(35.14)	74	1.05(0.66,1.69)	1.07(0.66,1.46)	7(5.56)	126	0.20(0.09,0.46	0.22(0.10,0.48)
Gender								
Male	52(30.95)	168	1.85 (1.32,2.62)*	1.44(0.97,2.14)	32(13.79)	232	0.80, (0.52,1.22)	0.97(0.64,1.49)
Female	47(16.67)	282			41(17.23)	238		
Previous hospitalization^###^								
Yes	20(44.44)	45	2.28(1.56,3.34)*	1.65(1.07,2.54)	6(23.08)	26	1.53(0.73,3.19)	
No	79(19.51)	405			67(15.09)	444		
Type of surgery					NA			
Elective	60(27.03)	222	1.58 (1.10, 2.26)*	0.98(0.65,1.46)				
Emergency	39(17.11)	228						
Type of wound #					NA			
Contaminated/ dirty	13(23.64)	55	1.30(0.77,2.22)	0.81(0.47,1.42)				
Clean contaminated	28(35.90)	78	1.98(1.35,2.89)*	1.28(0.85,1.94)				
Clean	56 (18.12)	309						
Recent endotracheal intubation								
Yes	24(19.83)	121	0.87(0.58,1.31)		2(40)	5	2.62(0.87,7.82)	
No	75(22.80)	329			71(15.27)	465		
Chest tube								
Yes	2(16.67)	12	0.75(0.21,2.70)		3(75.00)	4	5.05(2.75,9.26)*	4.14(2.30,7.46)
No	97(22.15)	438			69(14.84)	465		
Mechanical ventilator								
Yes	3(37.50)	8	1.72(0.69,4.29)		12(35.29)	34	2.52(1.51,4.20)*	1.99(1.06,3.74)
No	96(21.72)	442			61(13.99)	436		
Indwelling urinary catheter								
Yes	48(19.12)	251	0.74(0.53,1.05)		17(28.33)	60	2.07(1.29,3.32)*	1.28(0.76,2.14)
No	51(25.63)	199			56(13.66)	410		
IV line								
Yes	80(21.45)	373	0.87(0.56,1.34)		30(20.13)	149	1.50(0.98,2.29)	
No	19(24.68)	77			43(13.40)	321		
Naso-gastric tube								
Yes	3(9.68)	31	0.42(0.14,1.25)		5(25.00)	20	1.67(0.76,3.69)	
No	96(22.91)	419			67(14.32)	449		
Preoperative prophylaxis#					NA			
Given	88(22.11)	398	0.98(0.56,1.71)					
Not given	11(22.45)	49						
Underlying disease								
Yes	9(13.85)	65	0.59(0.31,1.11)		44(24.58)	179	2.54(1.64,3.93)*	2.01(1.33,3.04)
No	90(23.38)	385			28(9.66)	290		

*** Percentage was calculated from row total, *RR* relative risk, *ARR* Adjusted relative risk, *CI* – confidence interval, * Statistically significant # please note missing participant, ^###^ participants who had history of hospital admission in the past five years

Among surgical care participants, bi-variate analysis of risk ratio has indicated that male gender, having elective surgery, clean contaminated wound and history of previous hospitalization to predispose for HAIs (Table 2). However, the adjusted risk ratio analysis controlled for cofounders, stratified by age and gender has indicated that only participants with history of previous hospitalization had higher risk of developing HAIs (ARR = 1.65, 95% C.I:1.07, 2.54) (Table 2).

The mean age of participants with HAIs grouped under surgical care was 35.11 ± 20.07SD.This value is significantly higher than the mean age of the participants with no HAIs (31.09 ± 13.76SD, *t* = 0.022) in the same group. On the other hand multivariate adjusted risk ratio analysis has indicated that age group between 18 and 30 years had lower risk of developing HAIs (ARR = 0.54 95% C.I: 0.32,0.93)(Table 2).

Among non-surgical patients, bi-variate analysis of risk ratio has indicated that chest tube, indwelling urinary catheter, mechanical ventilation and underlying disease were the risk factors for HAIs. However, the multivariate analysis to depict adjusted risk ratio (ARR) indicated that chest tube (ARR = 4.14, 95% C.I: 2.30,7.46), mechanical ventilation (ARR = 1.99, 95% C.I: 1.06,3.74) and underlying diseases (ARR = 2.01, 95% C.I: 1.33,3.04) were only the risk factors for developing HAI (Table 2). The mean age of non surgical patients with HAI was 27.96 ± 16.44SD which is significantly lower than the mean age of participants with no HAI 36.52 ± 17.17, *t* = 0.0001. However, the risk of HAI is low between age 30 and 45 (ARR = 0.54, 95% C.I: 0.31,0.93), and among participants older than 45 years of age (ARR = 0.22, 95% C.I:0.10,0.48).

### HAI and discharge outcome

The mean duration of hospital stay for HAI patients was 13.95 days (SD ± 8.99) while that of patients without HAI was 7.63 days (SD ± 6.86). There is a significant mean difference of 6.32 days [(SE of difference = 0.59), 95% C.I: (5.16, 7.48), t = 0.000] between the two groups.

Overall, there were 44 deaths (in hospital mortality rate of 4.3%). The mortality rate in those with hospital acquired infection was 7.5%. Patients who developed HAI had 2.24 times increased risk of dying in the hospital (COR = 2.24, 95% CI:1.18–4.27, *P* = 0.014) than those who did not have it. This association persisted even after controlling for age, sex and underlying medical condition (AOR = 2.23, 95% CI: (1.15–4.29, *p* = .017).

## Discussion

The over all incidence rate of healthcare associated infection at JUMC is 28.15 per 1000 patient days. This is the first report for incidence rate of HAI from Ethiopia. It is higher than finding from Europe [32]. Our study also revealed an overall prevalence rate of HAI of 19.41%; a finding which is again much higher than most reports from high income settings [16, 20, 21]. It is also higher than the reported prevalence from some of the studies done in the country [25, 26]. This might be associated with the comprehensive nature of this study which involved all admitted patients including those in intensive care and those with debilitating medical conditions. These might have contributed for the observed higher prevalence.

When compared with other similar studies done in Africa, the finding of this study is higher than reports from Nigeria (2.6%), Morocco (10.3%) and Tanzania (14.8%) [33–35]. This could be explained by the high patient load, overcrowding, poor infrastructure and design of the hospital layout. On the other hand, the observed prevalence of HAI in this study is lower than reported prevalence from Addis Ababa (35.8%) and Mekelle (27.6%) [24, 27].

The incidence rate of HAI at different wards indicated a large variability that ranges from 0.98 per 1000 patient days at ophthalmology ward to 207.55per 1000 patient days at Intensive care unit (ICU). It is not surprising to see such high incidence of HAI at ICU given that most of the patients admitted to ICU are debilitated, critical and subjected to insertion of different medical devices. Such patients are at high risk of developing any infection especially in facilities with substandard infection prevention practice [36]. Studies in both low-and-middle, and high income countries also revealed the highest rates of HAI in ICU. Even in settings with better standards of care, HAI may affect up to half of patients in ICU [17]. Findings from other settings also revealed high prevalence of HAI in intensive care setting, 34.5% in Morocco and 46.7% from Saudi Arabia [34, 37].

In addition to patient and healthcare setting related factors, HAI is often related to surgical wounds and presence of indwelling devises [16]. As a result, urinary tract and surgical site infections remain the commonest forms [16, 24, 33]. In our study, UTI was found to be the commonest form of HAI followed by surgical site infection. Even though this is consistent with findings in different settings, the proportion of UTI in this study (68.71%) is higher than findings in the other studies [34, 38]. This might be associated with the diverse nature of our participants and high rate of urinary catheterization. Moreover, 50.85% of UTI were associated with indwelling urinary catheter.

Our finding has also revealed that the observed HAI infections are more of systemic infection than localized wound or soft tissue infection as evidenced by 27.69% of them developing two or more types of HAIs at the same time. Nevertheless, the overlap of multiple HAI in such patients deserves utmost attention due to the magnitude of the problem and threat to patient population getting service at the hospital. In line with this, the two most frequently isolated bacteria were *E.coli and Klebsella species.* These bacteria are known to cause community acquired UTI in Ethiopia [39, 40]. This finding suggests that these bacteria are also the main etiologist of nosocomial UTI and they might have colonized the medical devices or the hospital environment.

This study has indicated that among surgical care group the risk of HAI is higher on patients with history of previous hospitalization and lower in young adults (18–30 years). It is possible to speculate that patients who did visit hospitals frequently might have some chronic underlying disease which potentially expose them for HAIs. Furthermore, the possibility of colonozation by drug resistant bacteria in those with previous hospitalization might have also contributed to the occurrence of HAI in the current admission. On the other hand, the fact that young adults have better overall health status may expalin the lower risk of HAI in this group of patients.

The risk of HAI among non-surgical patients were higher on patients with chest tube, mechanical ventilator and those with underlying diseases. This result suggests most of HAIs in this group of patients related to contamination of the medical devices by bacteria. This can be due to improper handling and storage, and/or substandard application of aseptic procedures during handling, insertion and removal of the medical devices. This situation might also be further fueled by the existance of one or more underlying medical conditions.

Our study revealed a twofold increment in mortality in patients who developed HAI. Besides, six additional days of hospital stay in patients with HAI in this study is already a huge healthcare cost in any setting and deserves emergency action. As some of these patients might have also left the hospital setting without proper treatment, they risk potential dreadful outcome to themselves and dissemination of multidrug resistant strains to the community. This, in places where antibiotic choices are limited and health care seeking behaviour is low, may perpetuate a vicious circle that may affect the whole healthcare system unless kept in check urgently.

The strength of this study is that it included patients admitted to all wards of the hospital; prospectively followed them until discharge implementing proper technique for identification and susceptibility testing of causative bacteria. However, the full burden of HAI could not be captured as our study was limited to in hospital assessment leaving out patients who may potentially develop HAI after discharge. The second limitation is associated with failure to include neonatal ICU which may portray different nature of HAI to other types reported here. It should also be noted that our microbiological assessment was limited to bacterial HAI without considering other causes such as fungal infection. As this is the first comprehensive assessment to report incidence and prevalence of HAI at the hospital, we strongly believe that this finding may provide valuable imput to plan for proper intervention.

## Conclusion and recommendations

Hospital acquired infection is a significant problem at Jimma University Medical Centre. The problem is immense at ICU, Paediatrics and Surgical wards. Risk factors for HAI among patients who had surgery is different from those patients who did not have surgical intervention. Previous hospitalization history was the main independent risk factor for patients who had surgery. Whereas, insertion of medical devices and underlying diseases were the main risk factors for patients who did not have surgery. HAI was also associated with increased mortality and prolonged hospital stay. Hence, any prospective effort to control HAI at JUMC should consider this risk factor differences to tackle the problem with maximum positive outcome.

## Additional file

Additional file 1: Questioner. (PDF 77 kb)

## Abbreviations

BSI: Blood stream infection
HAI: Health care associated infection
ICU: Intensive care unit
JUMC: Jimma University Medical Center
NI: Nosocomial infection
SSI: Surgical site infection
UTI: Urinary tract infection
