# Supplementary material for: Healthcare associated infection and its risk factors among patients admitted to a tertiary hospital in Ethiopia: longitudinal study
Source: Antimicrob Resist Infect Control. 2018 Jan 5;7:2. doi: 10.1186/s13756-017-0298-5 (PMC5755436; doi:10.1186/s13756-017-0298-5)
Supplement: Additional file 1: — Questioner. (PDF 77 kb) [file 13756_2017_298_MOESM1_ESM.pdf]

# QUESTIONER FOR PATIENTS

1. Hospital number ____________________________________________________
2. Ward______________________________ Bed number ______________________
3. Age ___________________
4. Sex _____________________
5. Residence A. Urban___________ B. Rural____________
6. Occupation (care giver's in case of pediatric patients)
   - i. Government employee
   - ii. NGO employee
   - iii. Student
   - iv. Farmer
   - v. Merchant
   - vi. Daily laborer
   - vii. Others (specify)
7. Educational level (care giver's in case of pediatric patients)
   - i. Above first degree
   - ii. First degree
   - iii. Diploma
   - iv. High school
   - v. Elementary school
   - vi. Read & write
   - vii. illiterate
8. Marital status (skip for pediatric patients)
   - i. Married ii. Divorced iii. Widowed iv. Single
9. Monthly income (estimated)__________________________Birr
10. Complete admission diagnoses ____________________________________________
11. Previous hospitalization

    Yes ☐ No☐
12. If yes for question 11:

    A. Place (including ward) ________________________________________________

    B. Time (month/year) ___________________________________________________

    C. Duration___________________________________________________________
13. Previous antibiotic use for the current illness A. Yes__________ B. No_____________
14. If yes for question 13, specify______________________________________________
15. If yes for question 13, for how many days? ____________days
16. Being on mechanical ventilator? A. Yes_______ B. No___________
17. Presence of intravenous line? A. Yes_______ B. No___________

18. Presence of urinary catheters? A. Yes_______ B. No___________

19. Presence of invasive medical devices? A. Yes__________ B. No__________

20. If yes for questions 16-19 (more than one answer is possible)

    A. Endotracheal tube? A. Yes__________ B. No_________

    B. NGT A. Yes__________ B. No_________

    C. Chest tube A. Yes__________ B. No_________

21. Underlying diseases (more than one answer is possible)

| | |
|---|---|
| i. Diabetes mellitus | vi. Cardiac disorders |
| ii. Chronic renal failure | vii. Severe malnutrition |
| iii. Hypertension | viii. TB |
| iv. Chronic liver disease | ix. Cancer |
| v. HIV/AIDS | x. Others (specify) |

22. Surgical procedure done? A. Yes___________ B. No___________

23. If yes for question 22,

    A. Type of surgery A. Elective________ B. Emergency___________

    B. Type of the procedure____________________________________________________

    C. Date ________________ Time______________

    D. Duration of the surgery ________________hours

    E. Type of surgical wound A. Clean B. Clean contaminated C. Contaminated D. Dirty

24. Antibiotic prophylaxis given? A. Yes_____________ B. No____________

25. If yes for Q24, specify_____________________________________________________

26. If yes for Q24, how many doses? ____________________________________________

27. Stay in maternal waiting area for the index pregnancy A. Yes_______ B. No_______

28. If yes, how many days? ____________days

29. Any procedure done (including PV examination)? A. Yes__________ B. No_________

30. Duration of rupture of membrane____________

31. Duration of labor_______________________
